# Shunting in chronic hydrocephalus induces VEGFR-2 and blood vessel density changes in the caudate nucleus

**DOI:** 10.1186/1743-8454-7-S1-S55

**Published:** 2010-12-15

**Authors:** Mark Luciano, Stephen Dombrowski, Abhishek Deshpande, Natalie Krajcir, Jun Yang

**Affiliations:** 1 Section of Pediatric and Congenital Neurosurgery (S60); Department of Neurosurgery; Neurological Institute; Cleveland Clinic 9500 Euclid Avenue, Cleveland, OH 44195, USA

## Background

Chronic hydrocephalus (CH), which is characterized by increased cerebrospinal (CSF) fluid volume with or without increased intracranial pressure (ICP), is often associated with decreased cerebral blood flow and oxygen delivery. While CSF shunting can improve neurological symptoms, the cause of these symptoms in hydrocephalus and the mechanism of shunt reversal remain unclear. We have previously observed a decreased cerebral blood flow in CH which was associated with a stimulation of Vascular Endothelial Growth Factor Receptor- 2 (VEGFR-2) expression and changes in vascular density. In this study we investigated the effect of shunting on neuronal and glial VEGFR-2 expression and blood vessel densities (BVd) in the caudate nucleus.

## Materials and methods

Fourteen (n=14) young adult canines were divided into three groups: CH-Shunted (CH-S, shunted at 12 weeks, n=4); CH-Untreated hydrocephalic (CHU, 12-16 weeks, n=5); and Surgical Controls (SC, 12-16 weeks, n=5). The experimental model of CH used was previously developed and investigated in our lab. The density of blood vessels and VEGFR-2 positive neurons and glia was estimated using stereological counting methods and expressed as a percent (%) of total cells.

## Results

Untreated hydrocephalic animals had approximately 2-3 times the amount of %VEGFR-2 neurons compared to controls. Shunted animals had a significantly lower %VEGFR-2 neuronal expression (32%) compared to CH-U (50%) (p≤0.01). BVd was significantly lower in CH-U (826 BV/ mm3) and was lowest in CH-S (675 BV/mm3) compared to SC (1012 BV/ mm3; p≤0.05). %VEGFR-2 glial expression was not significantly different among the three groups. No correlation was found between %VEGFR-2, BVd and ventricular volume (VV); however, 3/4 animals showed a decrease in VV following shunting which was associated with improved BVd. Similarly, 2/4 animals showed improved oxygenation following shunting which was associated with improved BVd.

## Conclusions

In the caudate, CSF shunting appears to significantly reverse VEGFR-2 neuronal activation and CH-induced changes in blood vessel density. This is consistent with the hypothesis that hydrocephalus is associated with chronic hypoxia/ischemia which is resolved by CSF removal. Shunting does not seem to have any effect on VEGFR-2 glial expression which might suggest that other factors may be involved in glial activation. Control of VEGF system activation through agonists or antagonists may ultimately provide a means of reducing brain injury and improving function.

